# Fibrillin-1 mutant mouse captures defining features of human primary open glaucoma including anomalous aqueous humor TGF beta-2

**DOI:** 10.1038/s41598-022-14062-8

**Published:** 2022-06-23

**Authors:** MinHee K. Ko, Jeong-Im Woo, Jose M. Gonzalez, Gayeoun Kim, Lynn Sakai, Janos Peti-Peterdi, Jonathan A. Kelber, Young-Kwon Hong, James C. Tan

**Affiliations:** 1 Doheny Eye Institute, Los Angeles, CA USA; 2 Department of Medical and Molecular Genetics, Oregon Health Sciences University, Portland, OR USA; 3 Departments of Physiology, Biophysics and Medicine, Keck School of Medicine, University of Southern California, Los Angeles, CA USA; 4 Developmental Oncogene Laboratory, California State University Northridge, Northridge, CA USA; 5 Department of Surgery, Keck School of Medicine, University of Southern California, Los Angeles, CA USA; 6 Department of Ophthalmology, University of California Los Angeles, Los Angeles, CA USA; 7 Sightgene, Inc., 9227 Reseda Blvd, #182, Northridge, CA 91324-3137 USA

**Keywords:** Ocular hypertension, Optic nerve diseases

## Abstract

Primary open angle glaucoma (POAG) features an optic neuropathy, elevated aqueous humor (AH) TGFβ2, and major risk factors of central corneal thickness (CCT), increasing age and intraocular pressure (IOP). We examined Tight skin (Tsk) mice to see if mutation of fibrillin-1, a repository for latent TGFβ, is associated with characteristics of human POAG. We measured: CCT by ocular coherence tomography (OCT); IOP; retinal ganglion cell (RGC) and optic nerve axon counts by microscopic techniques; visual electrophysiologic scotopic threshold responses (STR) and pattern electroretinogram (PERG); and AH TGFβ2 levels and activity by ELISA and MINK epithelial cell-based assays respectively. Tsk mice had open anterior chamber angles and compared with age-matched wild type (WT) mice: 23% thinner CCT (*p* < 0.003); IOP that was higher (*p* < 0.0001), more asymmetric (*p* = 0.047), rose with age (*p* = 0.04) and had a POAG-like frequency distribution. Tsk mice also had RGCs that were fewer (*p* < 0.04), declined with age (*p* = 0.0003) and showed increased apoptosis and glial activity; fewer optic nerve axons (*p* = 0.02); abnormal axons and glia; reduced STR (*p* < 0.002) and PERG (*p* < 0.007) visual responses; and higher AH TGFβ2 levels (*p* = 0.0002) and activity (*p* = 1E−11) especially with age. Tsk mice showed defining features of POAG, implicating aberrant fibrillin-1 homeostasis as a pathogenic contributor to emergence of a POAG phenotype.

## Introduction

Primary open angle glaucoma (POAG)^1,2^ is an age-related optic neuropathy that is the leading cause of irreversible blindness worldwide. It is characterized by progressive loss of retinal ganglion cells, optic nerve axons and vision in association with major risk factors of intraocular pressure (IOP), age and central corneal thickness. It is not known if some overarching influence drives this constellation of features in POAG.

A further important but cryptic feature of POAG is elevated levels of transforming growth factor-β (especially TGFβ2) in the aqueous humor. This represents a biomarker that is replicable across many human ethnicities^3–13^. Elevated aqueous humor TGFβ is linked with altered biomechanical and fluid resistance properties of the trabecular meshwork (TM) and Schlemm’s canal^14–16^, a key aqueous humor drainage pathway determining IOP. Why aqueous humor TGFβ should be elevated in POAG is unclear.

Fibrillin-1 plays important roles in regulating the bioavailability and activity of TGFβ^17,18^. Fibrillin-1 is abundant in the extracellular matrix (ECM) where it serves as a backbone for elastic microfibrils and principal reservoir for latent TGFβ. TGFβ is secreted as a complex with latent TGFβ binding proteins (LTBP) that target TGFβ to fibrillin-1, forming a repository of latent TGFβ in the ECM. This raises an intriguing question of whether fibrillin-1 or microfibril anomaly in eye tissues could contribute to the aqueous humor TGFβ dysregulation of POAG.

Fibrillin-1 mutations cause Marfan syndrome^19^, an archetypal human disease in which cardiovascular, bony and eye problems^19,20^ are associated with abnormal TGFβ signaling^20,21^. Fibrillin-1 abnormality affects the functioning of elastic-rich organs such as heart, lung, blood vessels and skin (OMIM #154700, #608328). In the eye ectopia lentis develops wherein crystalline lens subluxation occurs due to weakness of fibrillin-1-rich lens zonules. Additionally, the prevalence of POAG is increased^22^. The TM is a richly elastic and expansile tissue with abundant elastic microfibrils^23,24^ supporting the eye’s drainage biomechanics and IOP regulation^25–28^. In human POAG, elastic microfibrils in the TM are degenerate^29^ and abnormal plaques containing fibrillin-1 form in the key aqueous humor drainage tissues of TM and inner wall of Schlemm’s canal^23,24^. This correlates with impaired drainage tissue elasticity^30^ and pulsatile outflow^25,26^ that is expected to feed a cycle of IOP dysregulation in POAG.

Microfibril function is affected not only by primary fibrillin-1 abnormality but also by mutations of proteins cooperating with fibrillin-1. Mutations of these cooperating proteins may yield a phenotype overlapping with that of primary fibrillin-1 mutation: an ADAMTS10 (a disintegrin and metalloproteinase with thrombospondin motifs) mutation in beagles causes POAG and ectopia lentis^31,32^; separate mutations of fibrillin-1 or ADAMTS10 cause an indistinguishable presentation of Weill Marchesani Syndrome in which eye abnormalities such as ectopia lentis coexist with brachydactyly, joint stiffness and short stature^33–35^; mutations of ADAMTS17 or LTBP2 cause a Weil Marchesani-like syndrome similar to that caused by fibrillin-1 and ADAMTS10 mutations^35,36^; and mutation of ADAMTSL4 (ADAMTS-like) causes an isolated ectopia lentis^37^. Furthermore, POAG is associated with mutations of fibrillin-1, ADAMTS10 and LTBP2^22,31,32,38,39^. These observations suggest a common disease pathway involving aberrant microfibril homeostasis.

The above considerations led us to explore the possibility that a POAG ocular phenotype develops in Tight skin (Tsk) fibrillin-1 mutant mice that have systemically impaired elasticity. Tsk mice are heterozygous for a spontaneous fibrillin-1 mutation^40^ that causes mutant (418 kDa) and wild-type (WT) fibrillin-1 (350 kDa)^41,42^ to coexist in tissues. This causes microfibril abnormality and elasticity derangements of major elastic tissues of lung, aorta and skin^42,43^. As the eye is also rich in elastic tissues, we wondered whether Tsk mouse eyes are affected as part of its systemic elastopathy and in a way that predisposes to TGFβ dysregulation and emergence of features resembling human age-related POAG.

## Results

**Figure 1:**
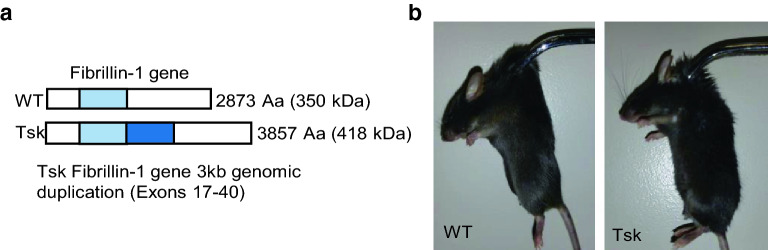
Tsk mouse mutation and skin phenotype. (**a**) Schematic of Tsk in-frame duplication mutation (darker blue, duplication of light blue region). (**b**) Decreased skin elastic compliance in Tsk (right) compared with WT (left) mouse.

We confirmed the genotype of our Tsk mice, which also had a stiff and inelastic skin phenotype (Fig. 1) characteristic of the mouse strain^41–43^.

### Thin central cornea and open anterior chamber angle configuration

**Figure 2:**
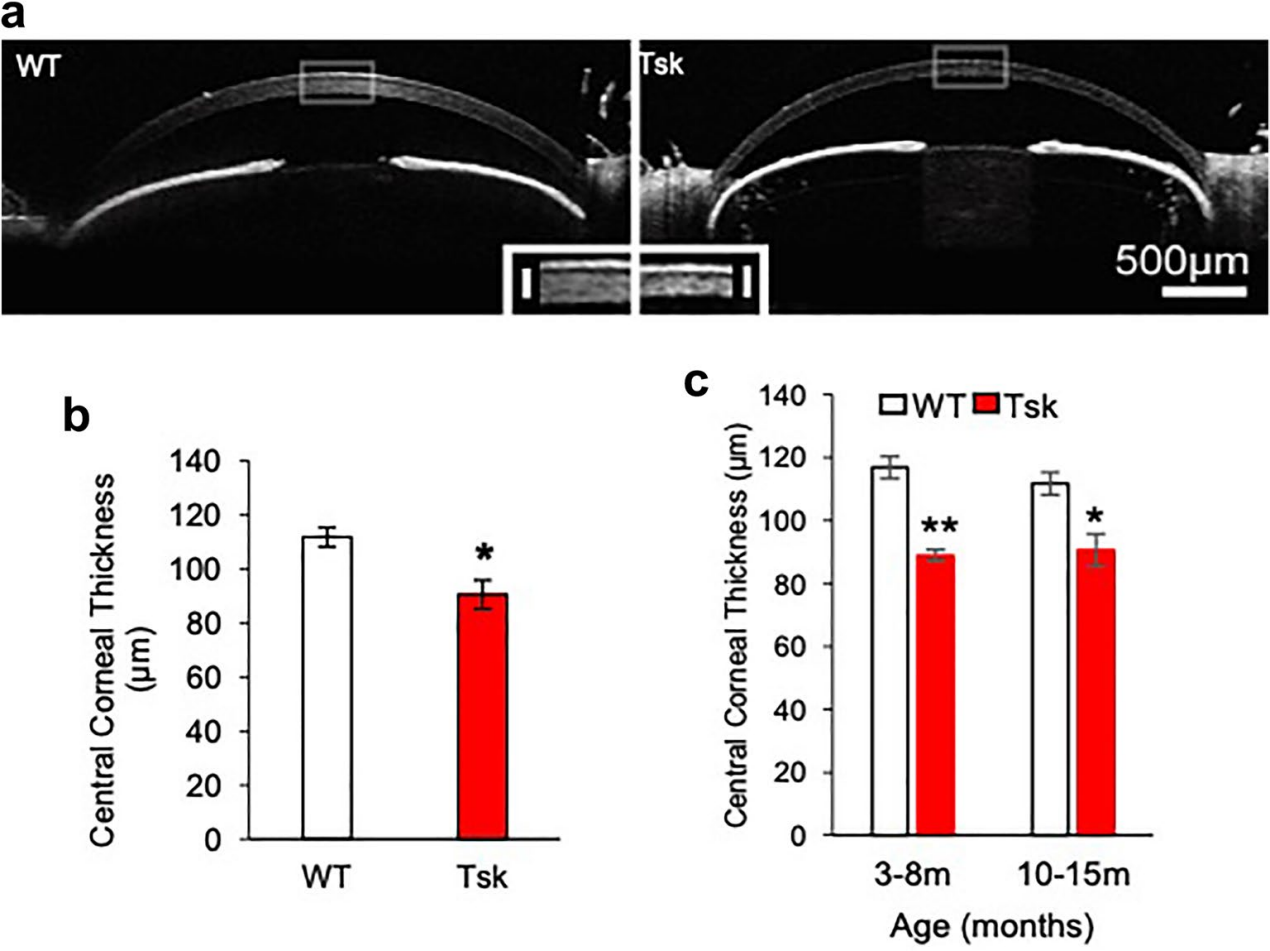
Tsk mouse central corneal thickness and anterior chamber angle configuration. (**a**) Ocular coherence tomography (OCT) images showing the cornea and anterior chamber configuration of wild-type (WT) and Tight skin (Tsk) mice. *Inset:* highlight of rectangular region of interest illustrating difference in central corneal thickness. (**b**) Comparison of central corneal thickness measurements between WT and Tsk mice (*p = 0.0003). (**c**) Central corneas were a mean of 23% thinner (**p = 7.7E−120) in younger Tsk (88.84 ± 4.85 µm; n = 7; mean ± standard deviation) than age-matched WT mice (116.85 ± 12.68 µm; n = 13; both aged 3–8 m). A similar difference was evident between older Tsk and age-matched WT mice aged 10–15 m (*p = 0.0003). *Bars:* mean; *Error bars:* standard error of mean.

Relatively thin central corneas are an independent risk factor for POAG and also associated with tonometric underestimation of true IOP^44–46^. Tsk mouse central corneas were 23% thinner compared with WT mice. Figure 2a, b respectively show this difference qualitatively (images) and quantitatively (*p* = 0.0003) based on ocular coherence tomography (OCT). The difference in central corneal thickness was already present in young mice aged 3–8 months and remained unchanged in older mice aged 10–15 months (Fig. 2c).

Anterior chamber angles of Tsk mice were open (Fig. 2a) at all ages, meeting a defining criterion of human POAG. Angle adhesions representing peripheral anterior synechiae that form in chronic angle closure configurations of glaucoma were not seen and did not develop with age.

### Validating tonometric intraocular pressure measurement

**Figure 3:**
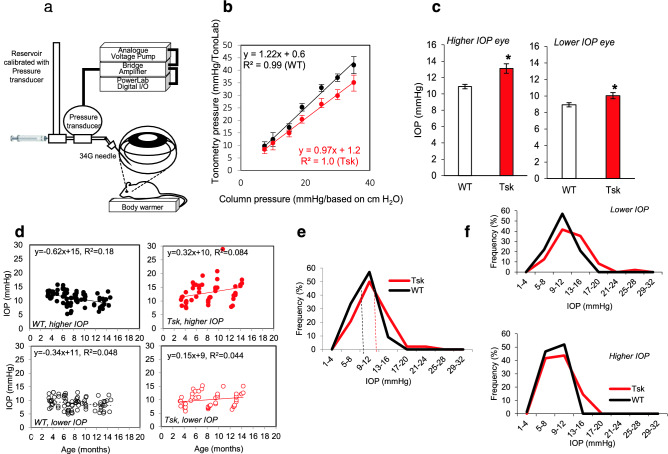
Tsk mouse IOP: validation, comparison and frequency distribution. (**a**) Illustration of perfusion apparatus for comparing Tonolab tonometry with pressure in the eye as established by hydrostatic pressure, constant pressure perfusion and simultaneous pressure transduction. (**b**) Relationship between rebound tonometry and hydrostatic pressure in the eye for Tsk (red) and WT mice (black). (**c**) Asymmetric IOP sorted as *higher IOP eyes* and fellow *lower IOP eyes* (left, *p = 0.0001; right, *p = 0.01). (**d**) IOP relationship with age (Tsk *higher IOP eyes (top right)*, *p* = 0.04; Tsk *lower IOP eyes (bottom right)*, *p* = 0.14; WT *higher IOP eyes (top left)*, *p* = 3.9E−05; WT *lower IOP eyes (bottom left); p* = 0.05). (**e**) Population frequency distribution of mean IOP (of fellow eyes) showing rightward skew toward higher IOP in Tsk (n = 48) relative to WT mice (n = 77). *Dotted lines:* mean IOP (Tsk, 11.6 mmHg; WT, 9.9 mmHg). (**f**) IOP population frequency distributions for *lower IOP eyes (top)* and *higher IOP eyes (bottom)*.

We tested if tonometric IOP measurements in Tsk and WT mice reflected ‘true pressure’ in the eye due to a possibility that inter-strain central corneal thickness differences may have influenced tonometry. Tonolab rebound tonometry measurement was performed under conditions of known intraocular hydrostatic pressure and constant pressure perfusion and confirmed by simultaneous pressure transduction for “true IOP” levels between 7 and 35 mmHg using a set up illustrated in Fig. 3a and described in detail in the Methods.

Scatter plots examining the relationship between rebound tonometry and “true pressure” had a linear distribution in Tsk and WT mouse eyes (R^2^ ≥ 0.99 for both; Fig. 3b). The linear regression model for Tsk mouse tonometry (y = 0.97x + 1.2; R^2^ = 1.0) indicated a roughly 1:1 rate of rise between tonometric readings and true pressure in the strain. The tonometric function for WT mouse eyes with thicker central corneas (y = 1.22x + 0.6; R^2^ = 0.99) was 26% steeper, however. This indicated a higher tonometric IOP rate of rise relative to true pressure in WT compared with Tsk mouse eyes. Strain-specific regression models were used to estimate ‘true pressure’ in the eye for each strain to avoid findings being biased by inter-strain central corneal thickness differences. This permitted valid inter-strain IOP comparisons.

### Higher intraocular pressure and age-related increase

IOP is a major risk factor for age-related human POAG and the only therapeutically modifiable risk factor. Both IOP level^45,47^ and IOP asymmetry^48–53^ confer risk of POAG.

We sorted IOP data from fellow eyes of each mouse into *higher IOP eye* and *lower IOP eye* groups to determine the extent of IOP asymmetry in Tsk and WT fellow eyes. Mean (± SD) IOP difference between fellow eyes reflecting asymmetry between *higher IOP eye* and *lower IOP eye* groups was 3.5 ± 2.4 mmHg in Tsk mice and 2.4 ± 2.9 mmHg in WT mice (difference, *p* = 0.047), indicating significantly greater IOP asymmetry between fellow eyes in Tsk compared with WT mice.

Figure 3c shows that IOP was higher in Tsk than WT mice in both *higher IOP eye* (Mean IOP 13.1 mmHg for Tsk; 10.9 mmHg for WT; *p* = 0.0001) and *lower IOP eye* groups (Mean IOP 10.0 mmHg for Tsk; 8.9 mmHg for WT; *p* = 0.012; Fig. 3c).

Figure 3d shows that Tsk mouse IOP was higher with age in the *higher IOP eye* group (*p* = 0.04) with a modeled IOP increase of 0.3 mmHg/month of age. The Tsk mouse *lower IOP eye* group also showed a trend toward higher IOP with age, although this was not significant (*p* = 0.14). Conversely WT mice showed significantly lower IOP with age in both *higher IOP eye* (*p* = 3.9E−05) and *lower IOP eye* groups (*p* = 0.05).

### Tsk intraocular pressure frequency distribution resembles human POAG

IOP frequency distribution analysis showed higher mean IOP and a longer right tail in Tsk relative to WT mice representing a rightward skew toward higher IOP in Tsk mice (Fig. 3e; Tsk, n = 48; WT, n = 77), as occurs in human POAG populations^54^. Mean IOP was higher in Tsk (11.6 mmHg) than WT (9.9 mmHg) mouse eyes. The same IOP frequency distribution pattern was present in *higher IOP eye* and *lower IOP eye* group comparisons between Tsk and WT mice (Fig. 3f).

### Retinal ganglion cell deficit and age-related decline

**Figure 4:**
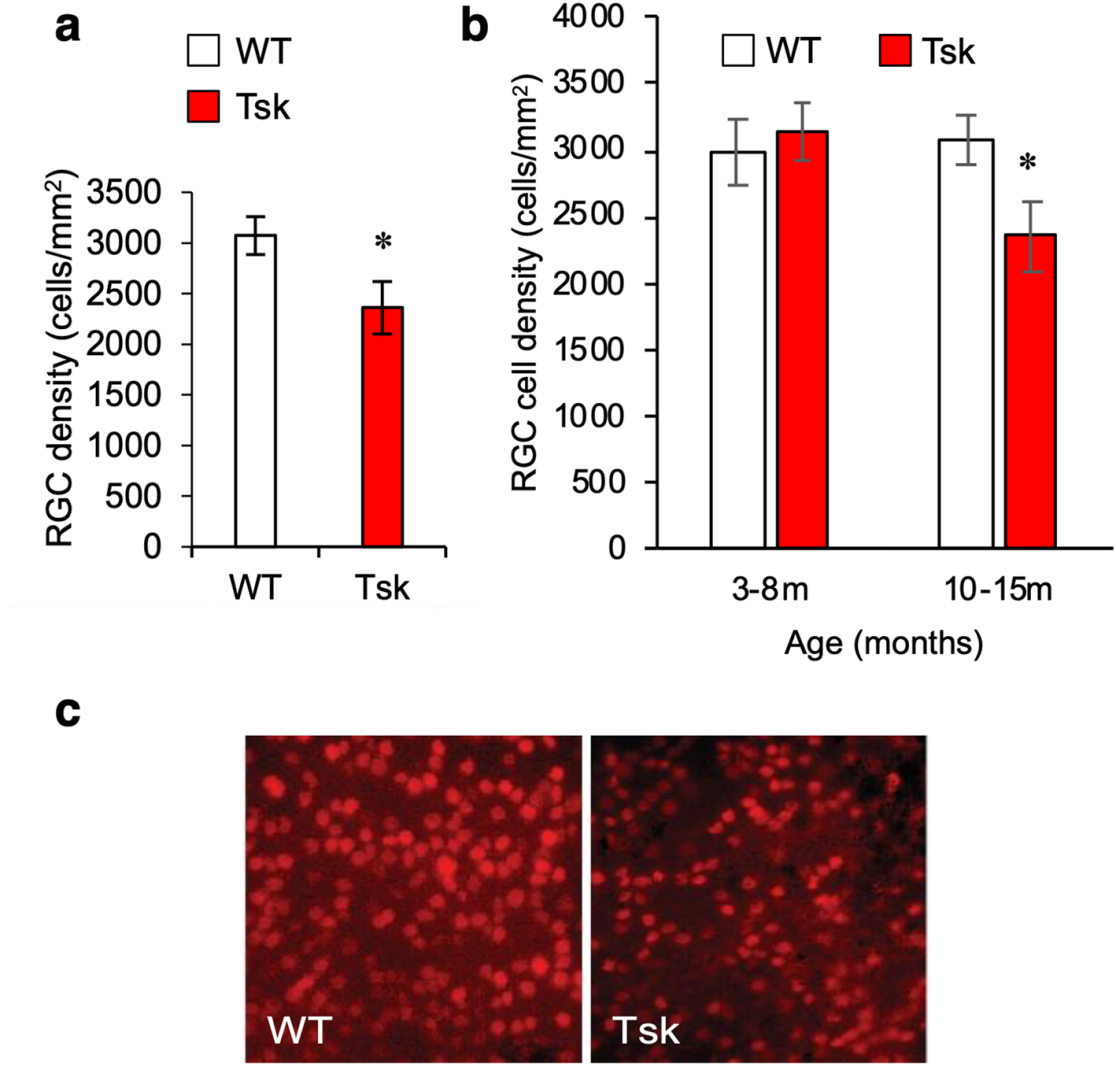
Reduced Tsk mouse RGC density. (**a**) RGC density in WT (n = 7) and Tsk mice (n = 9) aged 10–15 m (*p = 0.04). (**b**) RGC density in the retina of WT (n = 5) and Tsk (n = 6) mice aged 3–8 m was similar (*p* = 0.6) but when older at 10–15 m, RGC density had declined in Tsk (n = 9; *p = 0.03) but not WT (n = 7; *p* = 0.77) mice compared with younger counterparts. (**c**) Representative image of RGC (red) density in retinal whole-mounts (scale bar, 20 µm) based on BRN3a retinal labeling in Tsk (n = 6) and WT mice (n = 5) aged 10–15 m.

Retinal ganglion cell (RGC) labeling was performed to assess for the possibility of reduced RGC density, which is expected in animal experimental glaucoma and human POAG^55^. The retina of aged Tsk mice (10–15 m, n = 9) showed reduced RGC density compared with age-matched WT mice (10–15 m, n = 7; *p* = 0.04; Fig. 4a) and younger Tsk mice aged 3–8 m (*p* = 0.0003; Fig. 4b). Correspondingly sparser RGC distribution was evident in microscopy of retinal whole mounts from Tsk than WT mice aged 10–15 m (Fig. 4c).

**Figure 5:**
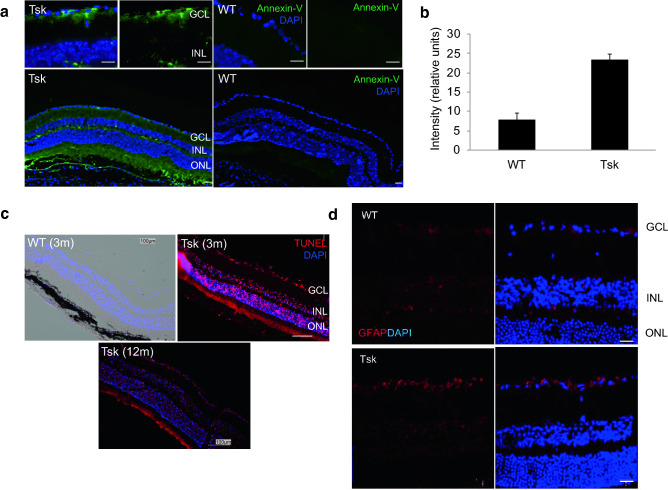
Increased Tsk mouse retinal apoptosis. (**a**) Representative images of apoptotic cell labeling (Annexin-V-FITC-positive; green) that was prominent in the GCL of Tsk (n = 5) but not WT (n = 5) mouse retina (aged 3–12 m; scale bar, 20 µm; top row). Annexin-V-positive labeling was also present in other retinal layers such as INL and ONL (bottom row). (**b**) Annexin-V labeling intensity was higher (*p* = 0.00005) in the GCL of Tsk (mean ± SD: 23.48 ± 2.73) than WT (7.87 ± 2.73) mouse retina. (**c**) Representative image showing TUNEL labeling (TUNEL positive, red) was higher in Tsk than WT mouse retina as early as 3 m and this was still present at 12 m of age (n = 3; scale bar: 20 µm). (**d**) Representative image of GFAP-positive labeling (red) indicating prominent glial activity in the GCL (RGC) layer of Tsk compared with WT mouse retina (scale bar, 20 µm; n = 5–6 per group).

Retinal Annexin-V-FITC (Fig. 5a,b) and TUNEL (Fig. 5c) labeling showed increased apoptosis in the ganglion cell layer (GCL) of Tsk compared with WT mice (*p* = 0.00005; Fig. 5b; age-matched 3–12 m). Additionally, indication of increased apoptosis was seen in the inner nuclear (INL) and outer nuclear layers (ONL) of Tsk compared with age-matched WT mice (Fig. 5a,c), which is not entirely surprising given outer portions of the retina may also be affected in glaucoma^56–61^. GFAP-positive labeling indicating prominent glial activity that occurs in glaucoma^62–65^ was increased in the GCL layer of Tsk compared with age-matched WT mice is shown in Fig. 5d.

### Visual functional abnormality

We performed visual electrophysiological testing to ascertain if functional deficit corresponding to the foregoing optic nerve and RGC changes was present in Tsk mice.

**Figure 6:**
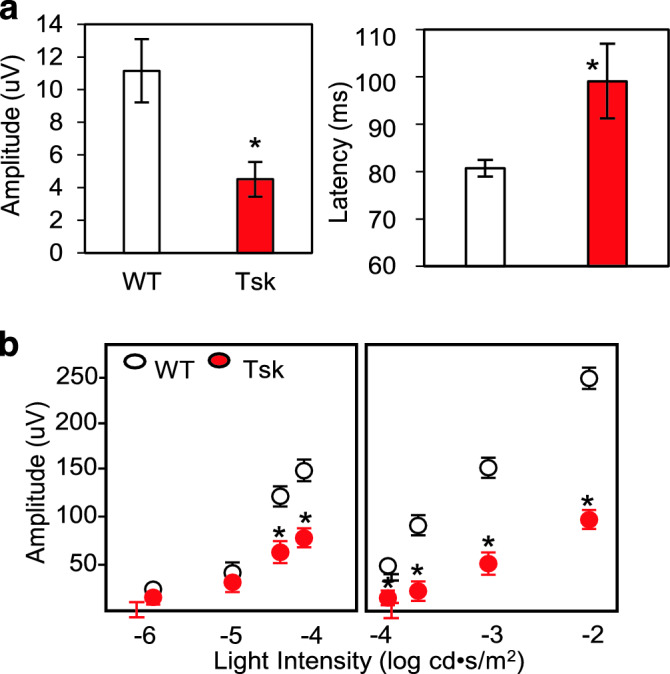
Abnormal Tsk mouse visual electrophysiology. (**a**) PERG (pattern electroretinogram) P1 wave amplitudes (*p = 0.006) and implicit time (*p = 0.04) in Tsk and WT mice. (**b**) pSTR (positive scotopic threshold responses) amplitudes (amplitude for − 4.4, *p = 0.0003; − 4.1, *p = 1E−05; − 4.0, *p* = 0.001; − 3.7, *p* = 3.9E−07; − 3.0 *p = E−05, and − 3.0, *p = 6E−05; stimulus: left, blue; right, white) in Tsk and WT mice. *Bars:* mean; *Error bars:* standard error of mean.

Pattern electroretinogram (PERG)^66–71^ testing showed that Tsk mice had 60% lower P1 amplitudes (*p* = 0.006) and 20% higher latency (*p* = 0.04) compared with age-matched WT mice (Fig. 6a).

Testing of positive scotopic threshold responses (pSTR)^72–75^ showed lower amplitudes (*p* < 0.001; Fig. 6b) in Tsk than WT eyes. These findings represent a functional correlate of inner retinal deficit that is typical of human POAG.

### Evidence of optic neuropathy

**Figure 7:**
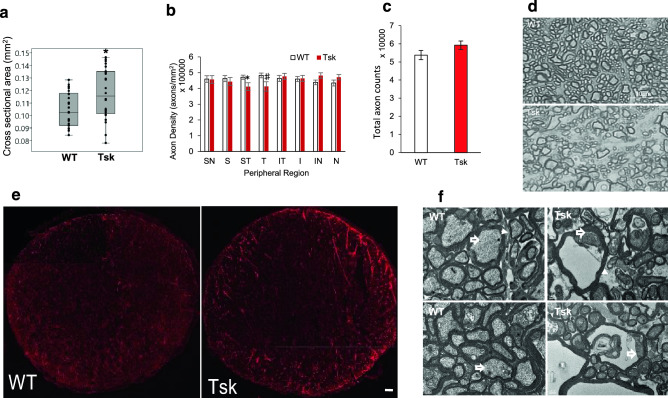
Tsk mouse optic neuropathy. (**a**) Optic nerve cross-sectional area was greater (*p = 0.01) in Tsk (n = 24) compared with WT mice (n = 22). (**b**) Analysis of axon density in optic nerve regions showed this was significantly lower (*p = 0.02, ♯*p* = 0.06; scale bar, 20 µm) superotemporally and possibly temporally. *S* = *superior; T* = *temporal; I* = *inferior; N* = *nasal. White bar: WT (n* = *8); red bar: Tsk (n* = *8)*. (**c**) Axon counts cumulative of all optic nerve sectors showed no difference (*p* = 0.98) between WT (n = 10) and Tsk mice (n = 8), masking specific regional differences seen in (**b**). (**d**) Looser packing and trend toward larger axons in the superotemporal region of Tsk (bottom) compared with WT (top) mouse optic nerves (both aged 11–14 m; paraphenylenediamine staining; magnification × 100; scale bar, 10 µm). (**e**) Glial activity in optic nerve seen in cross-section views (red, GFAP-`positive; scale bar, 20 µm) comparing Tsk and WT mice. (**f**) Altered organization of optic nerve axon myelin sheaths, neurofilaments (open arrows) and astrocyte processes (arrowheads; scale bar, 0.5 µm) of Tsk but not WT optic nerves in representative transmission electron microscopy. *Error bars:* standard error of mean; except 3A shows 2.5th to 97.5th centiles with median.

We determined if the foregoing IOP observations were associated with an optic neuropathy indicative of glaucoma. We found that optic nerve cross-sectional area was greater in Tsk than WT mice (*p* = 0.01; Fig. 7a), representing optic nerve expansion consistent with mouse early axonal degeneration and supporting the presence of an optic neuropathy in Tsk mice^72,76,77^.

Axon counting was performed in eighth sectors of optic nerve cross sections to identify possible regional axonal loss in Tsk (n = 7) relative to WT mice (n = 8). In sector-by-sector comparisons fewer axons were present in the superotemporal (ST; *p* = 0.02) and possibly temporal (*p* = 0.06) optic nerve sectors of Tsk compared with WT mice (Fig. 7b). Global counts obscured these more specific regional findings (Fig. 7c). Diminished axon counts were accompanied by altered axon organization (Fig. 7d) and heightened glial activity^62–65,76^ (Fig. 7e). Transmission electron microscopy (TEM) findings correlated with this in revealing variability of axon size, myelin sheath thinning, disordered axoplasm with irregular neurofilament arrangement, altered inter-axonal packing and glial process infiltration in Tsk but not WT mouse optic nerves (Fig. 7f).

### Aberrant trabecular meshwork elastic microfibrils

**Figure 8:**
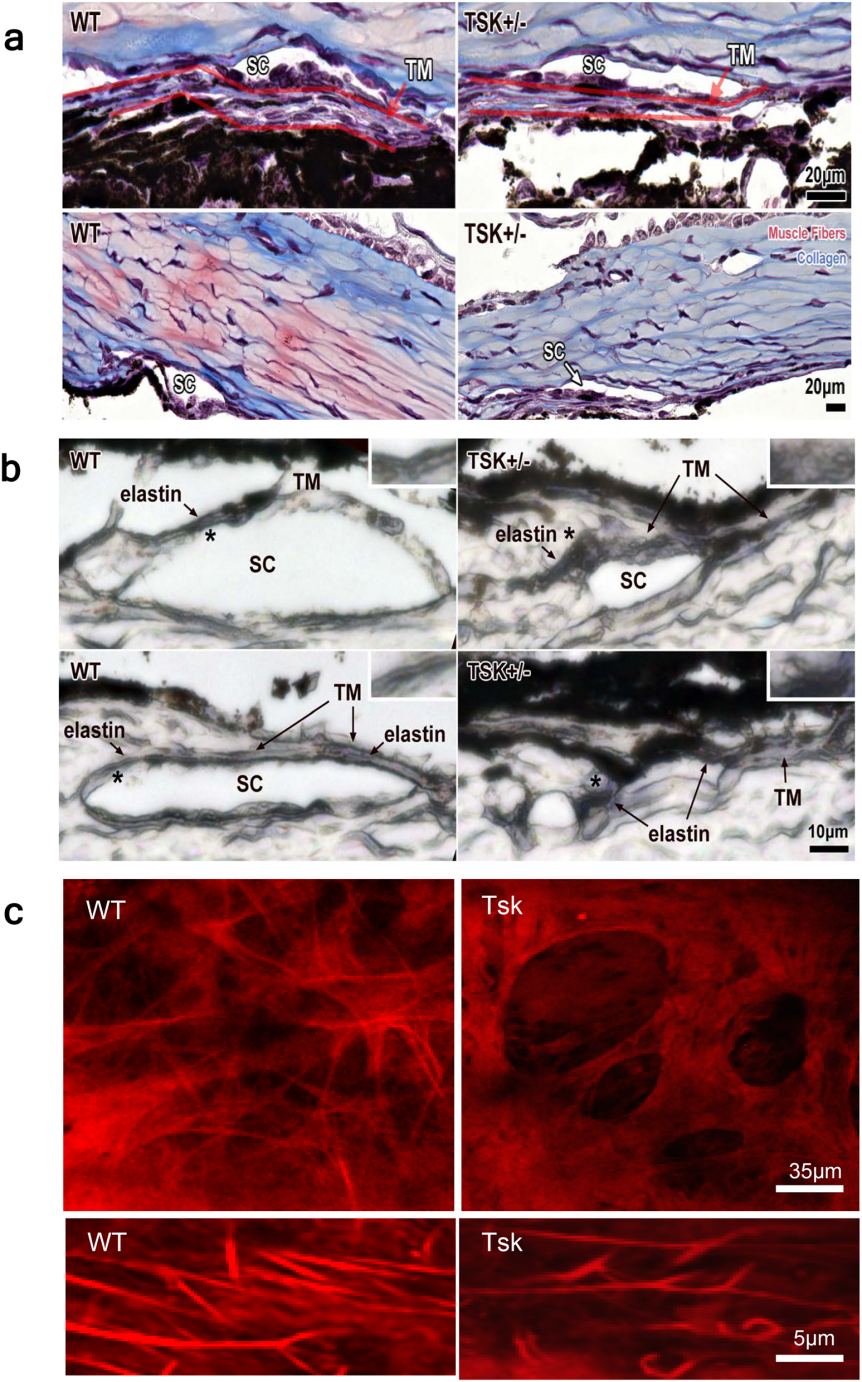
Tsk mouse elastopathy of trabecular meshwork. (**a**) Masson trichrome collagen staining of histologic sections of anterior chamber angle structures in Tsk and WT eyes (n = 6, scale bar, 20 um), *SC:* Schlemm’s canal. TM: trabecular meshwork. (**b**) Representative Verhoeff van Gieson elastin staining shows trabecular meshwork elastic microfibrils (*asterisks*) are curly in Tsk but linear in WT mice (n = 6 eyes each). *SC:* Schlemm’s canal. *Inset:* higher magnification. (**c**) 2-photon imaging of sulforhodamine-B-stained elastic microfibrils (bright red) in the beams (dim red) and pores (dark ovals) of the TM in enucleated eyes of Tsk and WT mice. *Bottom panel:* high magnification of elastic microfibrils.

We did not see gross histologic differences in the TM of Tsk compared with WT mice following H&E and Masson’s Trichrome staining to identify ECM collagen abnormality (Fig. 8a). Following Verhoeff van Gieson elastic staining of histologic cross sections, however, structural differences were notable as curly elastic microfibrils in the TM of Tsk mice (Fig. 8b). This compared with a more linear configuration of elastic microfibrils in the TM of WT mice (Fig. 8b). Similarly, 2-photon microscopy showed scant sulforhodamine-B-stained elastic microfibrils in beams and pores of the TM in Tsk compared with WT mice (Fig. 8c). Where elastic microfibrils were visualized in the TM of Tsk mice, they were sparse, fragmented and curled unlike the more abundant and linearly configured fibers that were prominent and densely distributed in the TM of WT mice.

### Increased aqueous humor TGFβ2 level and activity

**Figure 9:**
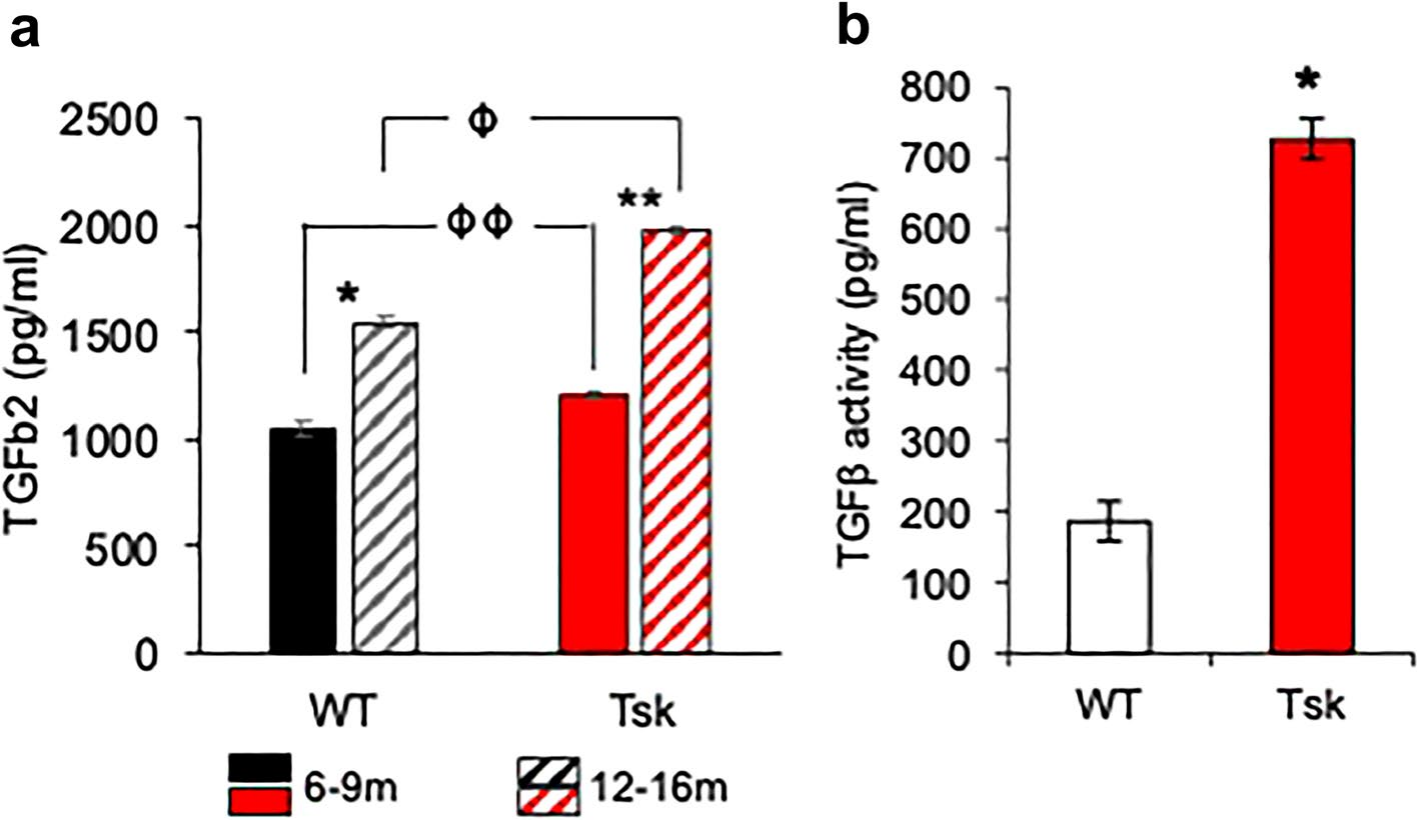
Tsk mouse anomalous aqueous humor TGFβ2. (**a**) Aqueous humor TGFβ2 levels in Tsk (red) and WT (black) mice aged 6–9 months (solid) and 12–16 months (striped) (n = 9–10 per group; based on duplicate assays, ELISA). *Comparisons:* * WT mice aged 6–9 m and 12–15 m (*p* = 0.006); ** Tsk mice aged 6–9 m and 12–15 m (*p* = 0.002); ϕϕ WT and Tsk mice aged 6–9 m (*p* = 0.07); ϕ WT and Tsk mice aged 12–15 m (*p* = 0.017). (**b**) Aqueous humor TGFβ2 activity in Tsk and WT mice (*p = 1E−11; Mink lung epithelial cell-based bioassay) (*p = 1E−11). *Bars:* mean; *Error bars*: standard error of mean.

Aqueous humor total TGFβ2 levels were higher in Tsk mice aged 12–16 months than Tsk mice aged 6–9 months (*p* = 0.006; Fig. 9a). Aqueous humor total TGFβ2 levels were also higher in WT mice aged 12–16 months than WT mice aged 6–9 months (*p* = 0.002; Fig. 9a). This indicates that TGFβ2 levels increased in the aqueous humor with age in both Tsk and WT mice. The rate of age-related increase was higher in Tsk (mean 61.1%) than WT mice (mean 49.9%), however (by 22.4%). Aqueous humor total TGFβ2 levels were higher in Tsk than age-matched WT mice at 12–16 months (by 48%; *p* = 0.0002) and possibly 6–9 months (by 27%, *p* = 0.07) of age.

Aqueous humor TGFβ2 activity, measured with a mink lung epithelial cell-based bioassay, was > 3.5 fold higher in Tsk than age-matched WT mice (*p* = 1E−11; Fig. 9b), resembling aqueous humor findings in human POAG^3–13,78^.

## Discussion

Tsk mouse eyes had an open anterior chamber angle configuration and displayed POAG risk factors of elevated and asymmetric IOP, thinner central corneas, and age-related features of higher IOP, lower RGC counts and elevated aqueous humor TGFβ2 levels with age. An optic neuropathy was present together with inner retinal dysfunction, declining RGCs and increased apoptosis in the GCL. The IOP frequency distribution pattern in our Tsk mouse population resembled that which is seen in human POAG populations^54^. Aberrant elastic fibers in the Tsk mouse TM were reminiscent of TM histologic abnormalities in human POAG^29^. Aqueous humor TGFβ2 levels and activity were increased, mimicking characteristics of this biomarker in human POAG^3–13,78^. Taken together Tsk mice carry a combined ocular clinical, biochemical and histopathologic age-related phenotype closely resembling that of human POAG. Our observations confirm and extend those of Kuchtey et al.^72^ who sought to identify glaucoma features in Tsk mouse eyes, raising intriguing questions about the contribution of fibrillin-1 or microfibril-related abnormality to emergence of a POAG phenotype.

Tsk mice showed various IOP characteristics that are relevant to the risk profile of human POAG in terms of level, asymmetry, age-relatedness and population frequency distribution. While there was a tendency for IOP to be higher in Tsk than WT mice, IOP in the strains broadly overlapped without a clearly definable cutoff by which presumed glaucoma could be defined. This mirrors observations in human populations where between 30 and 92% of subjects with POAG show IOP readings 21 mmHg or below^54,79–90^, a cut-off historically used to define elevated IOP but used more loosely these days. Significant IOP asymmetry was seen between fellow eyes and IOP was higher with age in the higher IOP eye group of Tsk mice. Here linear modeling indicated a 35% IOP increase between 3 and 15 months of age. A similar though insignificant trend was also present in the fellow lower IOP eye group. Notably, this age-related pattern was different from WT mouse eyes in which IOP was significantly lower with age. Additionally, we found that IOP was significantly higher in both higher IOP eye and lower IOP eye groups compared with WT mice.

That Kutchtey et al.^72^ did not find higher IOP in their Tsk mice as we did may relate to methodological differences. Firstly, we accounted for IOP asymmetry in the risk profile of POAG,^48–53^ and stratified data accordingly in our analyses. We also wanted to avoid missing subtle differences that might otherwise be obscured without appropriately segregating fellow eye data in the presence of IOP asymmetry. Secondly, we analyzed IOP frequency distribution and age-related trends in a sizable population of mice (Tsk, n = 48; WT, n = 77), which was necessary if we were to unearth patterns in the data. Thirdly, in the presence of significant inter-strain central corneal thickness differences, we felt it was necessary to validate tonometry readings by determinations of true pressure in the eye. Our validation models guided steps to ensure the validity of inter-strain IOP comparisons.

Tsk mouse RGC counts declined with age between 3–8 months (average 6 months) and 10–15 months (average 12 months). We did not detect a corresponding age-related RGC decline in WT mice. Significantly reduced RGC counts (mean ~ 25%) accompanied by increased apoptosis and glial activity in the GCL layer was present in Tsk mice aged 10–15 months. Why these findings differed from those of Kutchtey et al.^72^ is unclear. RGC loss may be patchy (61), and it may be that their sampling in discrete locations yielded different findings from our diffuse sampling over the entire retina. Like us, however, they found Tsk mouse pSTR and PERG electrophysiologic derangements, which are a functional correlate of inner retinal abnormality in mice with experimental glaucoma (for pSTR and PERG)^72–74^ and human POAG (for PERG)^66–71^.

Our optic nerve studies confirmed those of Kuchtey et al.^72^ who reported accelerated age-related optic nerve expansion and axon enlargement in Tsk mice. Like them, we did not find an obvious global difference in axon counts between Tsk and WT control mice; however, our exhaustive sector-by-sector optic nerve analysis revealed significant regional axon count abnormalities that were obscured in more global analyses. This was present in superotemporal-temporal regions of Tsk mouse optic nerves. Such regional findings are a well-recognized feature of human POAG, where localized superotemporal or inferotemporal nerve damage may be prominent. Tsk mouse regional axon loss was accompanied by ultrastructural abnormalities of axons, myelin sheaths and neuroflaments together with increased glial activity. These observations support the presence of an optic neuropathy in Tsk mice at or possibly before one year of age, with an age-related decline in RGC counts indicating the condition is progressive. Although the optic neuropathy was relatively early, visual functional loss was already present. Beyond this, it was hard to confirm subsequent neuropathic progression due to the strain’s limited longevity (15–16 months).

Elastic and expansile properties of the TM are important to facilitate a pulsatile aqueous outflow dynamic^25–28^. The finding of aberrant elastic microfibrils in the TM suggests altered elasticity in the Tsk mouse drainage tissues correlating with its systemic elastopathy. In human POAG, elastic degeneration^23,24,29^, decreased elasticity and increased ECM rigidity of the TM^30^ accompanies IOP dysregulation. Age-related IOP dysregulation in Tsk mice may reflect progressive elastic degeneration of the drainage tissues due to a fibrillin-1 defect, perhaps with some contribution of age.

The finding of elevated aqueous humor TGFβ2 levels and activity provides biochemical support for the notion of a POAG phenotype in Tsk mice. It also raises a possibility that the aqueous humor TGFβ2 anomaly of Tsk mice – and by inference POAG – relates to abnormal microfibril homeostasis. Several related observations support this idea: mutations of microfibril co-regulating families of fibrillin, LTBP, ADAMTS and ADAMTSL affect the microfibril microenvironment and can yield overlapping phenotypes^31–33^; different fibrillin-1 and LTBP2 mutations may cause an identical clinical phenotype^35^ and inappropriate TGFβ signaling^17,18^; separate mutations of fibrillin-1^22^, ADAMTS10^31,32^ and LTBP2^38,39^ are associated with POAG; and LTBP2 mutation is associated with POAG and pseudoexfoliation glaucoma^38,39,91–94^ that both show aqueous humor TGFβ anomalies. Could fibrillin-1 mutation disrupt latent TGFβ repositories in anterior segment tissues leading to anomalous aqueous humor TGFβ2? Does fibrillin-1 abnormality that structurally compromises elastic microfibrils^41,42,96–98^ affect the elastic eye tissues^29^ responsible for aqueous drainage biomechanics? These are intriguing questions awaiting further study for which the Tsk mouse could be useful.

## Methods

### Animal husbandry and anesthesia

Tsk mice (B6.Cg-Fbn1Tsk/J. stock #014632^40,72^ and C57BL/6J (stock # 00064) were purchased (The Jackson Laboratory) and bred in-house. Tsk homozygotes are embryonically lethal and we used Tsk heterozygotes mice for our study. Tsk mice and WT littermates as a control from the colony were used between 3 and 15 m of age. Both females and males were used in all experiments. All inter-strain comparisons involved age-matched mice. All experiments were in compliance with the ARVO Statement for Use of Animals in Ophthalmic and Vision Research and ARRIVE guidelines. Approval had been obtained from University of California, Los Angeles and California State University, Northridge Institutional Animal Care and Use Committees (IACUC) and all experiments were performed in accordance with relevant guidelines and regulations. The mice were raised and housed in air-filtered clear cages with a bedding of pine shavings, subject to a 12 h light/dark cycle, and fed ad libitum. Mice were anesthetized with a mixture of ketamine (85 mg/kg, Ketaject, Phoenix Pharmaceutical, Inc.), xylazine (8.5 mg/kg, AnaSed; Lloyd Laboratories) and acepromazine (2.125 mg/kg, Boehringer Ingelheim), injected intraperitoneally. Anesthesia was titrated to achieve a depth permitting ERG testing and aqueous humor collection. One drop of topical proparacaine hydrochloride ophthalmic solution (0.5%, Akorn Inc.) was applied to the cornea prior to experiments requiring ocular surface contact. Mice were rested on a warming platform or under a heating blanket to maintain body temperature during experiments.

### Genotyping Tsk mice by standard PCR

Tail tips (~ 1 mm) of each new pup were collected after weaning and genotyping was performed using standard PCR methods as recommended (The Jackson Laboratory). Briefly, three primers were synthesized (Integrated DNA Technologies Inc): 5′GGC TCC TTC CTC CCA CTT AG 3′ (WT); 5′ATC CCT GGG ACC ATA ACA CA 3′ (Common); 5′GAG TCC GAG TGT CCC TCA AG 3′ (Mutant). PCR was performed in a Mastercycler (Eppendorf) using a protocol of 1 cycle of 94 °C for 2 min (min), 10 cycles of 94 °C for 20 s (s), 65 °C for 15 s, and 68 °C for 10 s, and 28 cycles of 94 °C for 15 s, 60 °C for 15 s, and 72 °C for 10 s. Tsk heterozygotes (173 and 278 base-pair (bp)) and WT littermates (278 bp) were identified following 2% agarose gel electrophoresis.

### Central corneal thickness measurement

Relatively thin central corneas are an independent risk factor for POAG and associated with tonometric underestimation of true IOP^44–46^. Central corneal thickness (CCT) was measured in mice by ocular coherence tomography (OCT, Spectral Domain-OCT, Heidelberg engineering). Mice that were lightly sedated with intraperitoneal acepromazine (2 mg/kg; Boehringer Ingelheim) were positioned on an elevated platform and three scans were taken per eye and captured images were viewed in OCT software (Heidelberg Eye Explorer). CCT was determined by measuring the distance between the corneal epithelium and endothelium at the center of the cornea and on 4 more locations right next to center. Three independent investigators who were masked to mouse strain analyzed the images and conducted measurements in NIH ImageJ software^99^. Images were captured in Tsk and WT mice (aged 3–8 and 10–15 m; n = 5–13 mice per group).

### IOP measurements

IOP was measured by induction/impact rebound tonometry (TonoLab, ICare) 15 min after sedation with acepromazine intraperitoneally (2 mg/kg; Boehringer Ingelheim), as we have previously described^100^. Seven IOP readings were obtained in both eyes during the same morning session of each Tsk and WT mouse. The highest and lowest IOP values for each eye were discarded and remaining five IOP values were averaged^100^. IOP measurements were performed in mice aged 3–15 m (Tsk, n = 48; age 8.4 ± 3.6 m; WT, n = 77; age 8.2 ± 2.8 m).

### Validating tonometry measurement of IOP

Validation studies were performed in age-matched Tsk (n = 7) and WT (n = 11) mice aged 5–7 months to determine how closely IOP measurement by rebound tonometry approximated true pressure in the eye in each strain and if there were strain-specific differences to account for in subsequent analyses comparing IOP between Tsk and WT strains. This was important given the substantial and significant inter-strain central corneal thickness difference documented here and elsewhere^72^.

To validate rebound tonometry, mice were anesthetized and a 34-gauge needle was inserted just posterior to the corneoscleral limbus into the vitreous cavity of the eye (Fig. 3a). We avoided needle cannulation through the cornea to avoid biasing rebound tonometry readings. The needle was attached to a three-way connector connected to a suspended fluid reservoir at a height chosen to generate a pre-determined level of hydrostatic pressure. Reservoir height was serially varied in steps between 9.5 and 47.6 cm to change hydrostatically-determined pressure inside the eye (‘column pressure’) in a corresponding range of 7–35 mmHg. Hydrostatically-determined pressure in the eye was independently confirmed and tracked with a pressure transducer (P75; Hugo Sachs, March–Hugstetten, Germany). The pressure transducer was also connected via a second three-way connector with perfusion apparatus for constant-pressure perfusion that we have previously described^101,102^. Same-session constant pressure perfusion followed by rebound tonometry was performed to double check IOP validation based on hydrostatic pressure. One observer monitored hydrostatic pressure, constant pressure perfusion and transduced pressure in the eye while a separate independent observer measured IOP by rebound tonometry in the same mouse eye, as we and others have previously described^100,103^.

Pressure in the eye determined by hydrostatic means, constant pressure perfusion and pressure transduction represented ‘true pressure’ in the eye. Tonolab measurements were plotted against hydrostratic pressure to establish strain-specific linear regression models of Tonolab-hydrostatic pressure relationships. These models were used to ascertain true pressure in the eye from Tonolab readings for each strain that were used for all inter-strain comparisons. This approach allowed valid IOP comparisons between strains and a way to circumnavigate the influence of inter-strain central corneal thickness differences that is well known to bias tonometry^44,45^.

### Analysis of inter-strain IOP differences

IOP, which is a major risk factor for POAG, was analyzed in Tsk and WT mice along the lines of IOP asymmetry, level, age-relatedness and population frequency distribution that have been used to characterize risk of POAG in humans:

- *IOP asymmetry* IOP difference between eyes of each mouse was calculated by subtracting the lower IOP value of one eye from the higher IOP value of the fellow eye. Difference values for each mouse were pooled for each strain and analyzed. Larger inter-eye IOP differences represented greater IOP asymmetry between fellow eyes. Pooled IOP differences were compared between strains to ascertain the extent of inter-eye IOP asymmetry in Tsk and WT mice.
- *IOP level with asymmetry* IOP measurements from fellow eyes of each Tsk and WT mouse were sorted into two groups: the higher IOP reading was separated into a group of *higher IOP eyes* and the lower IOP reading into a group of *lower IOP eyes* for each mouse strain. *Higher IOP eye group* data were compared between Tsk and WT mice. Likewise, *lower IOP eye group* data were compared between the strains.
- *IOP age-relatedness* IOP values for fellow *higher IOP eye* and *lower IOP eye* groups were plotted by mouse age for Tsk and WT mice. Linear regression analysis was performed to characterize trends in IOP level that might be seen with age for each mouse strain.
- *IOP frequency distribution* To describe the IOP distribution in Tsk and WT mouse populations, the number of mice/eyes having IOP within different IOP ranges (i.e., 1–4, 5–8, 9–12, 13–16, 17–20, 21–24, 25–28, 29–32 mmHg) was determined. The percentage of total population represented by mouse/eye numbers for the IOP ranges was calculated and plotted as frequency distribution curves for *higher IOP eye*, *lower IOP eye,* and *mean IOP* (of both eyes) groups of Tsk and WT mice.

### Retinal whole mount preparation and RGC labeling

RGC labeling was performed to identify reduced RGC density,^104,105^ which occurs in experimental glaucoma and human POAG^55–60^. Eyes were enucleated and retinas were dissected and prepared as flattened whole mounts by making 4–6 radial cuts followed by post-fixing in 4% paraformaldehyde for an additional hour and rinsing in PBS. Primary antibody labeling was performed with goat anti-BRN3a antibody (1:50 dilution, Cat# sc31984, Santa Cruz) at 4 °C overnight followed by 1 h incubation in blocking solution (5% BSA and 0.01% Triton X-100 in PBS). After washing with PBS, the tissue was incubated with donkey anti-goat 568 secondary antibody (Invitrogen) for 1 h at RT and mounted ganglion cell side up on slides in anti-fade DAPI mounting media (Vector labs). Retinas were photographed using a fluorescence microscope with 20 × objective (KEYENCE microscope). A total of 12 images were captured to cover an entire retinal whole-mount. Images were analyzed for BRN3a-positive RGC cells using NIH ImageJ software. RGC were counted in 4 ROI per image. For validation automated computer-aided RGC counts were compared with manual RGC counts by three independent, masked operators and found to agree. n = 7 mice aged 10–15 m were used in each group.

### RGC apoptosis

Apoptotic RGCs were detected using a TACS Annexin V-FITC apoptosis detection kit (Cat#4830-01-K, R&D Systems) and TUNEL in situ cell death detection kit (Cat#12 156792910, TMR Red, Roche). Eye cryosections and retinal whole mounts were incubated with Annexin V-FITC stock solution (1:10 ratio) for 30 min at RT or TUNEL for 60 min at RT according to manufacturer’s instructions. Sections were examined immediately by fluorescence microscopy (KEYENCE microscope) then covered with mounting medium and coverslip. DNase-treated sections served as positive controls. Additional tissue sections were incubated in binding buffer as negative controls.

### Immunohistochemistry and quantitative analysis

Enucleated eyes were fixed with 4% paraformaldehyde overnight and embedded in Tissue-Tek OCT compound (VWR). Cryosections (7 µm thickness) were permeabilized/blocked (5% bovine serum albumin (BSA), 0.3% Triton X-100, 1 h at RT). Sections from n = 6 mice aged 10–14 m per group were incubated overnight at 4 °C with mouse anti-GFAP (Cat# MAB360, Chemicon) primary antibody in blocking solution. The sections were further incubated for 1 h at RT with Alexa Fluor® 568-conjugated anti-mouse secondary antibody (Invitrogen) and then mounted using ProLong Gold Anti-fade reagent with 4',6-diamidino-2-phenylindole (DAPI, Thermo Scientific). Presence of non-specific labeling was established with normal IgG isotypes. Sections were analyzed on a Zeiss LSM 710NLO confocal microscope. Immunofluorescence staining was performed in randomly selected slides (4–5 slides per eye) containing 4 sections per slide. Specific fluorescence from tissue labeling in histological sections was captured by confocal microscopy with exposure time kept constant across all images. Image sections were imported as 16-bit images and analyzed in NIH ImageJ software using methods for analyzing intensity in regions of interest we have described^106–108^.

### ERG pSTR and PERG testing

Mice aged 10–13 m (Tsk, n = 11; WT, n = 10) were dark-adapted overnight, placed on a platform (Celeris, Diagnosys LLC) with heater (temperature of 99°F (37 °C) ± 3%) and prepared for ERG under red LED (center wavelength 660 and 940 nm). Pupils were dilated with topical atropine (0.5%) and phenylephrine (2.5%) and corneas anesthetized with topical proparacaine (0.5%). A contact lens paired with corneal electrode (Ag/AgCl) was applied to the mouse cornea with intervening hypromellose lubricating and coupling gel solution (0.3%, Allergan). Impedance for every test was in the range of 6–12 kΩ.

pSTR^72–75^ were tested using the company’s TOUCH/TOUCH protocols (Celeris), stimulating one eye at a time, with the fellow, unstimulated eye serving as a reference control so that both eyes were tested in alternating sequence. pSTR were tested at stimulus intensities of − 6.0, − 5.0, − 4.4, − 4.1, − 4.0, − 3.7, − 3.0, and − 2.0 log cd∙s/m^2^ (2000 Hz frequency; 50 ms sweep pre-trigger time; 300 ms sweep post trigger time; 10 sweeps per result; 6000 ms first sweep delay; and 500 ms inter sweep delay). Blue light stimuli were presented for stimulus intensities of − 6.0 to − 4.1 log cd∙s/m^2^ and white light stimuli were presented for stimulus intensities of − 4.0 to − 2.0 log cd∙s/m^2^. Low (0.0125 Hz) and high (300 Hz) frequency cut-off filters were set for each channel.

PERG^66–71^ was performed using a pattern stimulator electrode (Celeris) on the right eye of each mouse using the manufacturer’s program. A pattern light guide electrode presented a black/white contrast pattern screen through the corneal electrode (luminance range of up to 850Cd/m^2^; − 0.1 cd∙s/m^2^; 2000 Hz frequency). Visual function was determined by analyzing the a- and b-wave pattern.

### Optic nerve and axon manual quantification

For our cross-sectional analysis of optic nerve cross-sectional area across the colony, images (0.015 mm^2^ per image: x = 108 µm, y = 144 µm) were taken per optic nerve section at 100× magnification with an oil-immersion lens (n = 8–10 mice per group; age 3–6 m and 11–14 m). 1–2 images covered the center and 4–8 images covered the periphery of temporal, superotemporal, superior, superonasal, nasal, inferonasal, inferior, and inferonasal regions of the optic nerve. Images of peripheral optic nerve regions covered the nerve edge. Each image was subdivided into 64 ROI (242.5µm^2^ per ROI), from which 16 ROI were randomly selected to be manually counted by two individuals masked to mouse strain. Criteria for identifying axons were agreed upon, guided by TEM images. Cross-sectional area of optic nerves was calculated in NIH ImageJ (20X magnification)^74^. Two perpendicular lines were drawn edge to edge to mark the shortest and longest axis of the optic nerve. These were measured and converted to µm units. Half the average of the two axes was taken to represent the radius of a circle and used to calculate optic nerve cross-sectional area (Tsk, n = 24; WT, n = 22; age 3–14 m). Axon counts were divided by cross-sectional area to calculate axon density in different regions around the optic nerve in older mice (Tsk, n = 8; WT, n = 10; age 11–14 m; axons/mm^2^).

### Optic nerve paraphenylenediamine (PPD) staining and transmission electron microscopy (TEM)

After the mouse skull was cut open, optic nerves were dissected from underneath the brain, marked for orientation and cut 2 mm posterior to the nerve-globe junction. Optic nerves were fixed in ½ K buffer overnight and post-fixed in 2% osmium tetroxide for 2 h, dehydrated in a graded ethanol series, and embedded in resin. Sections (1 µm thick) were cut and stained with 1% PPD^75,76^. Bright field images were acquired with a 100× objective lens on a microscope (Keyence microscopes). For TEM, ultrathin cross-sections (70 nm thickness) of optic nerve (n = 6 mice/group, age 3–6 m and 11–14 m) were counterstained with 1% uranyl acetate and Sato lead and placed on formvar-coated grids. The sections were viewed with an electron microscope (model 2100 EM, JEOL).

### Elastin staining

To identify elastic microfibrils, elastin staining was performed using a Verhoeff van Gieson Elastic Stain Kit (Cat# HT25A, Sigma) in whole eye cross-sections according to the manufacturer’s protocol. Images were captured and analyzed (KEYENCE microscope).

For two-photon imaging, ex vivo mouse eyes were labeled intact, without dissection or sectioning, with Sulforhodamine-B (Thermo Fisher Scientific), a water soluble stain of elastic microfibrils. Microscopy was performed on a Leica SP5 microscope (Leica Microsystems, Heidelberg, Germany) coupled to a Chameleon Ultra-II multiphoton laser (Coherent, Santa Clara, CA) through inverted 20X/0.7NA or 63X/1.3NA objectives. We used 850 nm excitation, pulsed, and focused through green (525/50 nm) or red (585/40 nm) filters (Chroma, Bellows Falls, VT) onto a non-descanned photomultiplier tube detector (Hamamatsu, Bridgewater, NJ). Images were collected as multiple channel z-stacks using 512 × 512 or 1024 × 1024 pixel frames, 16-bit grayscale resolution, and 16X line averaging using 1–8 μm step sizes. Images were viewed and processed on LAS AF and AF Lite 2.2.1 (Leica), Image J (NIH; Bethesda, MD), Photoshop CS5 (Adobe, San Jose, CA) and Imaris (Oxford Instruments). Methods reported here have previously been described^101, 106–108^.

### Mouse aqueous humor preparation

Aqueous humor was collected from both eyes of each anesthetized mouse using anterior chamber perfusion apparatus under a dissecting microscope^101,102^. A 35-gauge needle (5 mm long, Medicom, Canada) connected to Hamilton syringe (10µL with luer tips) on a micromanipulator (MM33 Rechts, Germany) was used to cannulate the anterior chamber through the cornea without disrupting adjacent tissues. Protein quantification (BCA protein assay kit, Thermo Scientific) was performed after adding protease inhibitor cocktail (Calbiochem).

### ELISA for TGFβ2 in aqueous humor

Total TGFβ2 levels were measured in mouse aqueous humor (Tsk and age-matched WT mice, ages 6–9 m and 10–15 m; n = 20–22 mice per group) using a TGFβ2 ELISA detection kit (mouse, Cat# MB200, R&D Systems). Each standard protein was serially diluted in triplicates. Samples in triplicate were prepared from two independent sets of aqueous humor pooled from a minimum of 6 mice each. TGFβ in the samples was activated with 1 N HCl (1:2 dilution; 10 min at RT) then neutralized with 1.2 N NaOH/0.5 M HEPES according to the manufacturer’s protocol.

### Measurement of active TGFβ in aqueous humor

TGFβ activity was determined by a quantitative bioassay using CCL-64 mink lung epithelial cells (MLEC). MLEC were a generous gift from Dr. Daniel B. Rifkin, Professor, NYU School of Medicine. MLEC had been permanently transfected with p800neoLUC containing a truncated PAI-1 promoter fused to the firefly luciferase reporter gene that is extremely sensitive to TGFβ-dependent upregulation of PAI-1^109^. Briefly, MLEC were grown in DMEM containing 10% fetal bovine serum (FBS), 1X Penicillin–streptomycin-L-glutamine, and 250 µg/mL Geneticin (Thermo Scientific). Detached MLEC with 0.05% Trypsin–EDTA (Thermo Scientific) were re-plated in a 96-well plate (3.5 × 10^4^ cells/well) and incubated for 3–4 h. Cells were further incubated (16–20 h; 37 °C) in triplicate with pooled aqueous humor from Tsk and WT mice aged 6–9 m (n = 12 each group, for 2 independent experiments) diluted 1:7 in DMEM without FBS. Optimal aqueous humor concentrations were pre-determined by serial dilutions. Assays were referenced to TGFβ2 standards in triplicate (0.12–800 pM range). MLEC were lysed with lysis buffer (Analytical Luminescence) to release the luciferase reporter protein. 30μL of the cell extract were transferred to a white 96-well plate to which was added 100µL of luciferin buffer (200 mM tricine, 1.07 mM (MgCO_3_)_4_ Mg(OH)_2_, 2.7 mM MgSO_4_, 0.1 mM EDTA, and 33.3 mM DTT) containing 800 µM luciferin and 750 µM ATP to permit emitted light measurement over 3 s in a luminometer (SpectraMax® iD5 Microplate reader, Molecular Device). Luciferase activity was recorded as relative light units (RLU) and the RLU values were converted to TGFβ activity (pg/ml) using a TGFβ2 standard curve. Controls included samples with neutralizing anti-TGFβ2 antibodies.

### Statistics

Hypothesis testing was performed using two-tailed Students t-tests unless otherwise specified. The Mann–Whitney test was used when data distribution was non-parametric. Results were presented in the text as the mean ± standard deviation (SD). Data was presented in graphs as the mean with error bars for standard error of mean (SEM). Boxplots showed the median, 25th and 75th centiles and error bars for 2.5th and 97.5th centiles. Statistical analysis and graphing were performed in software packages, Past 3.0 (Paleontological Statistics Software Package; University of Oslo, Norway) and Excel for Mac 2011 software (Microsoft Corp). Asterisks (*) in graphs indicated *p* ≤ 0.05 representing statistical significance, with exact *p*-values noted in the corresponding figure legend.

## Data Availability

All data generated or analyzed during this study are included in this published article.

## References

[1] Tan JCH, Kaufman PL, Yanoff M, Duker J (2014). Primary open angle glaucoma. Yanoff & Duker's Ophthalmology.

[2] Weinreb RN, Khaw PT (2004). Primary open-angle glaucoma. Lancet.

[3] Tripathi RC, Li J, Chan WF, Tripathi BJ (1994). Aqueous humor in glaucomatous eyes contains an increased level of TGF-beta 2. Exp. Eye Res..

[4] Inatani M (2001). Transforming growth factor-beta 2 levels in aqueous humor of glaucomatous eyes. Graefes Arch. Clin. Exp. Ophthalmol..

[5] Ochiai Y, Ochiai H (2002). Higher concentration of transforming growth factor-beta in aqueous humor of glaucomatous eyes and diabetic eyes. Jpn. J. Ophthalmol..

[6] Picht G, Welge-Luessen U, Grehn F, Lütjen-Drecoll E (2001). Transforming growth factor beta 2 levels in the aqueous humor in different types of glaucoma and the relation to filtering bleb development. Graefes Arch. Clin. Exp. Ophthalmol..

[7] Lutjen-Drecoll E, Shimizu T, Rohrbach M, Rohen JW (1986). Quantitative analysis of ‘plaque material’ in the inner- and outer wall of Schlemm’s canal in normal- and glaucomatous eyes. Exp. Eye Res..

[8] Ozcan AA, Ozdemir N, Canataroglu A (2004). The aqueous levels of TGF-beta2 in patients with glaucoma. Int. Ophthalmol..

[9] Yamamoto N, Itonaga K, Marunouchi T, Majima K (2005). Concentration of transforming growth factor beta2 in aqueous humor. Ophthalmic Res..

[10] Min SH, Lee TI, Chung YS, Kim HK (2006). Transforming growth factor-beta levels in human aqueous humor of glaucomatous, diabetic and uveitic eyes. Korean J. Ophthalmol..

[11] Trivedi RH, Nutaitis M, Vroman D, Crosson CE (2011). Influence of race and age on aqueous humor levels of transforming growth factor-beta 2 in glaucomatous and nonglaucomatous eyes. J. Ocul. Pharmacol. Ther..

[12] Stefan C (2008). TGF-beta2 involvements in open angle glaucoma (Romanian). Oftalmologia.

[13] Takai Y, Tanito M, Ohira A (2012). Multiplex cytokine analysis of aqueous humor in eyes with primary open-angle glaucoma, exfoliation glaucoma, and cataract. Invest. Ophthalmol. Vis. Sci..

[14] Gottanka J, Chan D, Eichhorn M, Lütjen-Drecoll E, Ethier CR (2004). Effects of TGF-beta2 in perfused human eyes. Invest. Ophthalmol. Vis. Sci..

[15] Nakamura Y (2002). Signaling mechanism of TGF-beta1-induced collagen contraction mediated by bovine trabecular meshwork cells. Invest. Ophthalmol. Vis. Sci..

[16] Fleenor DL (2006). TGFbeta2-induced changes in human trabecular meshwork: implications for intraocular pressure. Invest. Ophthalmol. Vis. Sci..

[17] Handford PA, Downing AK, Reinhardt DP, Sakai LY (2000). Fibrillin: from domain structure to supramolecular assembly. Matrix Biol..

[18] Ramirez F, Rifkin DB (2009). Extracellular microfibrils: contextual platforms for TGFbeta and BMP signaling. Curr. Opin. Cell Biol..

[19] Sakai LY, Keene DR, Renard M, De Backer J (2016). FBN1: The disease-causing gene for Marfan syndrome and other genetic disorders. Gene.

[20] Neptune ER (2003). Dysregulation of TGF-beta activation contributes to pathogenesis in Marfan syndrome. Nat. Genet..

[21] Habashi JP (2006). Losartan, an AT1 antagonist, prevents aortic aneurysm in a mouse model of Marfan syndrome. Science.

[22] Izquierdo NJ, Traboulsi EI, Enger C, Maumenee IH (1992). Glaucoma in the Marfan syndrome. Trans. Am. Ophthalmol. Soc..

[23] Rohen JW, Lutjen-Drecoll E, Flugel C, Meyer M, Grierson I (1993). Ultrastructure of the trabecular meshwork in untreated cases of primary open-angle glaucoma (POAG). Exp. Eye Res..

[24] Ueda J, Yue BY (2003). Distribution of myocilin and extracellular matrix components in the corneoscleral meshwork of human eyes. Invest. Ophthalmol. Vis. Sci..

[25] Johnstone M, Martin E, Jamil A (2011). Pulsatile flow into the aqueous veins: manifestations in normal and glaucomatous eyes. Exp. Eye Res..

[26] Johnstone MA (2004). The aqueous outflow system as a mechanical pump: evidence from examination of tissue and aqueous movement in human and non-human primates. J. Glaucoma.

[27] Khatib TZ (2019). Hemoglobin video imaging provides novel in vivo high-resolution imaging and quantification of human aqueous outflow in patients with glaucoma. Ophthalmol. Glaucoma.

[28] Huang AS (2017). Aqueous angiography in living nonhuman primates shows segmental, pulsatile, and dynamic angiographic aqueous humor outflow. Ophthalmology.

[29] Chi HH, Katzin HM, Teng CC (1957). Primary degeneration in the vicinity of the chamber angle; as an etiologic factor in wide-angle glaucoma: II. Am. J. Ophthalmol..

[30] Last JA (2011). Elastic modulus determination of normal and glaucomatous human trabecular meshwork. Invest. Ophthalmol. Vis. Sci..

[31] Kuchtey J (2013). Screening ADAMTS10 in dog populations supports Gly661Arg as the glaucoma-causing variant in beagles. Invest. Ophthalmol. Vis. Sci..

[32] Kuchtey J (2011). Mapping of the disease locus and identification of ADAMTS10 as a candidate gene in a canine model of primary open angle glaucoma. PLoS Genet..

[33] Sengle G (2012). Microenvironmental regulation by fibrillin-1. PLoS Genet..

[34] Faivre L (2003). Clinical homogeneity and genetic heterogeneity in Weill-Marchesani syndrome. Am. J. Med. Genet A.

[35] Haji-Seyed-Javadi R (2012). LTBP2 mutations cause Weill-Marchesani and Weill-Marchesani-like syndrome and affect disruptions in the extracellular matrix. Hum. Mutat..

[36] Morales J (2009). Homozygous mutations in ADAMTS10 and ADAMTS17 cause lenticular myopia, ectopia lentis, glaucoma, spherophakia, and short stature. Am. J. Hum. Genet..

[37] Ahram D (2009). A homozygous mutation in ADAMTSL4 causes autosomal-recessive isolated ectopia lentis. Am. J. Hum. Genet..

[38] Jelodari-Mamaghani S (2013). Contribution of the latent transforming growth factor-beta binding protein 2 gene to etiology of primary open angle glaucoma and pseudoexfoliation syndrome. Mol. Vis..

[39] Saeedi O (2018). Delineation of novel compound heterozygous variants in LTBP2 associated with juvenile open angle glaucoma. Genes.

[40] Siracusa LD (1996). A tandem duplication within the fibrillin 1 gene is associated with the mouse tight skin mutation. Genome Res.

[41] Gayraud B, Keene DR, Sakai LY, Ramirez F (2000). New insights into the assembly of extracellular microfibrils from the analysis of the fibrillin 1 mutation in the tight skin mouse. J. Cell Biol..

[42] Green MC, Sweet HO, Bunker LE (1976). Tight-skin, a new mutation of the mouse causing excessive growth of connective tissue and skeleton. Am. J. Pathol..

[43] Akita M, Lee SH, Kaneko K (1992). Electron microscopic observations of elastic fibres in the lung and aorta of tight-skin and beta-aminopropionitrile-fed mice. Histol. Histopathol..

[44] Brandt JD, Beiser JA, Kass MA, Gordon MO (2001). Central corneal thickness in the Ocular Hypertension Treatment Study (OHTS). Ophthalmology.

[45] Gordon MO (2002). The ocular hypertension treatment study: baseline factors that predict the onset of primary open-angle glaucoma. Arch. Ophthalmol..

[46] Copt RP, Thomas R, Mermoud A (1999). Corneal thickness in ocular hypertension, primary open-angle glaucoma, and normal tension glaucoma. Arch. Ophthalmol..

[47] Wilson RM, Martone JF, Ritch R, Shields MB, Krupin T (1996). Epidemiology of chronic open-angle glaucoma. The Glaucomas.

[48] Lee AJ, Rochtchina E, Mitchell P (2004). Intraocular pressure asymmetry and undiagnosed open-angle glaucoma in an older population. Am. J. Ophthalmol..

[49] Levine RA (2006). Asymmetries and visual field summaries as predictors of glaucoma in the ocular hypertension treatment study. Invest. Ophthalmol. Vis. Sci..

[50] Ocular Hypertensive Treatment Study Group and European Glaucoma Prevention Study Group (2008). The accuracy and clinical application of predictive models for primary open-angle glaucoma in ocular hypertensive individuals. Ophthalmology.

[51] Crichton A (1989). Unequal intraocular pressure and its relation to asymmetric visual field defects in low-tension glaucoma. Ophthalmology.

[52] Cartwright MJ, Anderson DR (1988). Correlation of asymmetric damage with asymmetric intraocular pressure in normal- tension glaucoma. Arch. Ophthalmol..

[53] Williams AL (2013). The value of intraocular pressure asymmetry in diagnosing glaucoma. J. Glaucoma.

[54] Iwase A (2004). The prevalence of primary open-angle glaucoma in Japanese: the Tajimi Study. Ophthalmology.

[55] Kerrigan LA (1997). TUNEL-positive ganglion cells in human primary open-angle glaucoma. Arch. Ophthalmol..

[56] Cuenca N (2010). Changes in the inner and outer retinal layers after acute increase of the intraocular pressure in adult albino Swiss mice. Exp. Eye Res..

[57] Panda S, Jonas JB (1992). Innere Körnerschicht der Retina in Augen mit sekundärem Winkelblockglaukom (Inner nuclear layer of the retina in eyes with secondary angle-block glaucoma). Ophthalmologe.

[58] Panda S, Jonas JB (1992). Decreased photoreceptor count in human eyes with secondary angle-closure glaucoma. Invest. Ophthalmol. Vis. Sci..

[59] Nork TM (2000). Swelling and loss of photoreceptors in chronic human and experimental glaucuomas. Arch. Ophthalmol..

[60] Bayer AU (2001). Retinal morphology and ERG response in the DBA/2NNia mouse model of angle-closure glaucoma. Invest. Ophthalmol. Vis. Sci..

[61] Mittag TW (2000). Retinal damage after 3 to 4 months of elevated intraocular pressure in a rat glaucoma model. Invest. Ophthalmol. Vis. Sci..

[62] Oikawa K (2020). Sub-region-specific optic nerve head glial activation in glaucoma. Mol. Neurobiol..

[63] Bosco A, Steele MR, Vetter ML (2011). Early microglia activation in a mouse model of chronic glaucoma. J. Comp. Neurol..

[64] Ebneter A, Casson RJ, Wood JPM, Chidlow G (2010). Microglial activation in the visual pathway in experimental glaucoma: spatiotemporal characterization and correlation with axonal injury. Invest. Ophthalmol. Vis. Sci..

[65] Yuan L, Neufeld AH (2001). Activated microglia in the human glaucomatous optic nerve head. J. Neurosci. Res..

[66] Pérez de Lara MJ (2014). Assessment of inner retina dysfunction and progressive ganglion cell loss in a mouse model of glaucoma. Exp. Eye Res..

[67] Porciatti V, Saleh M, Nagaraju M (2007). The pattern electroretinogram as a tool to monitor progressive retinal ganglion cell dysfunction in the DBA/2J mouse model of glaucoma. Invest. Ophthalmol. Vis. Sci..

[68] Parisi V (2006). Clinical ability of pattern electroretinograms and visual evoked potentials in detecting visual dysfunction in ocular hypertension and glaucoma. Ophthalmology.

[69] North RV (2010). Electrophysiological evidence of early functional damage in glaucoma and ocular hypertension. Invest. Ophthalmol. Vis. Sci..

[70] Pinto LH (2007). Interpretation of the mouse electroretinogram. Doc. Ophthalmol..

[71] Porciatti V (2007). The mouse pattern electroretinogram. Doc. Ophthalmol..

[72] Wu HJ, Hazlewood RJ, Kuchtey J, Kuchtey RW (2018). Enlarged optic nerve axons and reduced visual function in mice with defective microfibrils. eNeuro.

[73] Heiduschka P, Julien S, Schuettauf F, Schnichels S (2010). Loss of retinal function in aged DBA/2J mice: new insights into retinal neurodegeneration. Exp. Eye Res..

[74] Perez de Lara MJ (2014). Assessment of inner retina dysfunction and progressive ganglion cell loss in a mouse model of glaucoma. Exp. Eye Res..

[75] Saszik SM, Robson JG, Frishman LJ (2002). The scotopic threshold response of the dark-adapted electroretinogram of the mouse. J. Physiol..

[76] Cooper ML (2016). Early astrocyte redistribution in the optic nerve precedes axonopathy in the DBA/2J mouse model of glaucoma. Exp. Eye Res..

[77] Calkins DJ (2012). Critical pathogenic events underlying progression of neurodegeneration in glaucoma. Prog. Retin Eye Res..

[78] Agarwal P, Daher AM, Agarwal R (2015). Aqueous humor TGF-beta2 levels in patients with open-angle glaucoma: a meta-analysis. Mol. Vis..

[79] Sommer A (1991). Relationship between intraocular pressure and primary open angle glaucoma among white and black Americans. The Baltimore Eye Survey. Arch. Ophthalmol..

[80] He M (2006). Prevalence and clinical characteristics of glaucoma in adult Chinese: a population-based study in Liwan District, Guangzhou. Invest. Ophthalmol. Vis. Sci..

[81] Xu L (2004). The prevalence and its screening methods of primary open angle glaucoma in defined population-based study of rural and urban in Beijing. Zhonghua Yan Ke Za Zhi.

[82] Mitchell P, Smith W, Attebo K, Healey PR (1996). Prevalence of open-angle glaucoma in Australia: the Blue Mountains Eye Study. Ophthalmology.

[83] Wensor MD (1998). The prevalence of glaucoma in the Melbourne visual impairment project. Ophthalmology.

[84] Shen SY (2008). The prevalence and types of glaucoma in Malay people: the Singapore Malay eye study. Invest. Ophthalmol. Vis. Sci..

[85] Bourne RR (2003). Prevalence of glaucoma in Thailand: a population-based survey in Rom Klao District, Bangkok. Br. J. Ophthalmol..

[86] Dielemans I (1994). The prevalence of primary open-angle glaucoma in a population-based study in The Netherlands: the Rotterdam study. Ophthalmology.

[87] Bonomi L (1998). Prevalence of glaucoma and intraocular pressure distribution in a defined population. The Egna-Neumarkt Study. Ophthalmology.

[88] Klein BE (1992). Prevalence of glaucoma: the Beaver Dam eye study. Ophthalmology.

[89] Quigley HA (2001). The prevalence of glaucoma in a population-based study of Hispanic subjects: Proyecto VER. Arch. Ophthalmol..

[90] Varma R (2004). Prevalence of open-angle glaucoma and ocular hypertension in Latinos: the Los Angeles Latino Eye Study. Ophthalmology.

[91] Kuehn MH (2016). A mutation in LTBP2 causes congenital glaucoma in domestic cats (felis catus). PLoS ONE.

[92] Narooie-Nejad M (2009). Loss of function mutations in the gene encoding latent transforming growth factor beta binding protein 2, LTBP2, cause primary congenital glaucoma. Hum. Mol. Genet..

[93] Desir J (2010). LTBP2 null mutations in an autosomal recessive ocular syndrome with megalocornea, spherophakia, and secondary glaucoma. Eur. J. Hum. Genet..

[94] Krumbiegel M (2009). Exploring functional candidate genes for genetic association in german patients with pseudoexfoliation syndrome and pseudoexfoliation glaucoma. Invest. Ophthalmol. Vis. Sci..

[95] Ali M (2009). Null mutations in LTBP2 cause primary congenital glaucoma. Am. J. Hum. Genet..

[96] Ashworth JL (1999). Fibrillin degradation by matrix metalloproteinases: implications for connective tissue remodelling. Biochem. J..

[97] Hindson VJ (1999). Fibrillin degradation by matrix metalloproteinases: identification of amino- and carboxy-terminal cleavage sites. FEBS Lett..

[98] Kuo CL (2007). Effects of fibrillin-1 degradation on microfibril ultrastructure. J. Biol. Chem..

[99] Schneider CA, Rasband WS, Eliceiri KW (2012). NIH Image to ImageJ: 25 years of image analysis. Nat. Methods.

[100] Chu ER (2014). Intraocular pressure measurement in acepromazine-sedated mice. Clin. Exp. Ophthalmol..

[101] Ko MK, Yelenskiy A, Gonzalez JM, Tan JC (2014). Feedback-controlled constant-pressure anterior chamber perfusion in live mice. Mol. Vis..

[102] Ko MK, Kim EK, Gonzalez JM, Tan JC (2016). Dose- and time-dependent effects of actomyosin inhibition on live mouse outflow resistance and aqueous drainage tissues. Sci. Rep..

[103] Wang WH, Millar JC, Pang IH, Wax MB, Clark AF (2005). Noninvasive measurement of rodent intraocular pressure with a rebound tonometer. Invest. Ophthalmol. Vis. Sci..

[104] Danias J (2003). Quantitative analysis of retinal ganglion cell (RGC) loss in aging DBA/2NNia glaucomatous mice: comparison with RGC loss in aging C57/BL6 mice. Invest. Ophthalmol. Vis. Sci..

[105] Salinas-Navarro M (2009). Retinal ganglion cell population in adult albino and pigmented mice: a computerized analysis of the entire population and its spatial distribution. Vis. Res..

[106] Chu ER, Gonzalez JM, Tan JC (2014). Tissue-based imaging model of human trabecular meshwork. J. Ocul. Pharmacol. Ther..

[107] Gonzalez JM (2017). Deep tissue analysis of distal aqueous drainage structures and contractile features. Sci. Rep..

[108] Gonzalez JM (2017). Toward in vivo two-photon analysis of mouse aqueous outflow structure and function. Exp. Eye Res..

[109] Abe M (1994). An assay for transforming growth factor-beta using cells transfected with a plasminogen activator inhibitor-1 promoter-luciferase construct. Anal Biochem..

